# Severe Gastrointestinal Bleed Caused by a Rectal Dieulafoy Lesion

**DOI:** 10.7759/cureus.20672

**Published:** 2021-12-24

**Authors:** Ranbir Singh, Harsh Patel, Bhavin M Patel, Franklin E Kasmin

**Affiliations:** 1 Internal Medicine, NewYork-Presbyterian Brooklyn Methodist Hospital, Brooklyn, USA; 2 Gastroenterology, NewYork-Presbyterian Brooklyn Methodist Hospital, Brooklyn, USA; 3 Gastroenterology, Aventura Hospital and Medical Center, Aventura, USA

**Keywords:** acute blood loss anemia, acute blood loss, gastrointestinal bleeding, colonoscopy, dieulafoy lesion

## Abstract

Dieulafoy lesions are vessels that erode the overlying epithelium without the presence of an ulcer. When these lesions bleed, they can frequently be self-limited, but bleeding can be recurrent and prolonged. Although most commonly found in the lesser curvature of the proximal stomach, there are reports of these lesions in other gastrointestinal tract regions. This case identifies a Dieulafoy lesion found in the rectum, which was the source of this patient’s profuse rectal bleeding.

## Introduction

A Dieulafoy lesion is a dilated aberrant submucosal vessel that erodes the overlying epithelium without a primary ulcer. The caliber of the artery is in the range of 1-3 mm, which is approximately 10 times the average caliber of mucosal capillaries. Most of these lesions are within the proximal stomach along the lesser curvature [1]. The etiology of a Dieulafoy's lesion is unknown, and events that trigger bleeding episodes are not well explained in current literature. Common risk factors include patients with chronic kidney disease, alcohol abuse, and cardiovascular disease. There have been rare case reports of these lesions found in other areas of the gastrointestinal (GI) tract, including the esophagus, duodenum, and colon. This case report presents a patient with severe lower gastrointestinal bleeding with a Dieulafoy lesion in the rectum.

## Case presentation

An 87-year-old gentleman with a past medical history of hypertension and hyperlipidemia was initially presented to the emergency department by Emergency Medical Services after being found on the ground unresponsive. The patient had been admitted for acute kidney injury and severe hypernatremia. Hemoglobin upon admission was 20.1 g/dL. This value was likely hemoconcentrated as after receiving intravenous fluid, his hemoglobin remained on an average of 13.1 g/dL throughout the rest of his hospital course.

**Figure 1:**
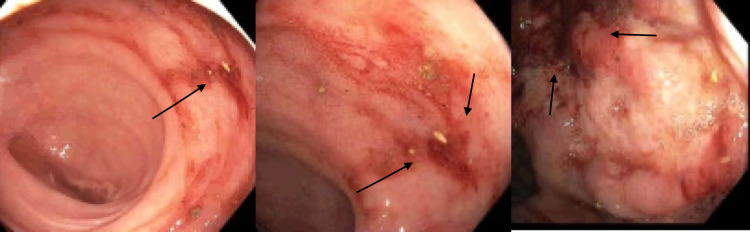
Dieulafoy lesion located at the rectum during flexible sigmoidoscopy as indicated by arrows

**Figure 2:**
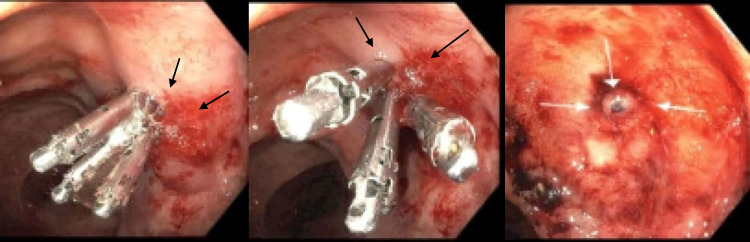
Dieulafoy lesion with three hemostatic clips (black arrows). The bleb presents the status post-injection of 6cc 1:10,000 solution of epinephrine (white arrows).

On hospital day 10, the floor staff activated a rapid response for hypotension and severe bright red bleeding per rectum and was hypotensive. Hemoglobin collected at that time was 9.5 g/dL. The patient received 500 mL of normal saline and three packed red blood cell units and was upgraded to the medical intensive care unit. The intensive team consulted the gastroenterology team, and they did an urgent flexible sigmoidoscopy. Findings were significant for a nonbleeding Dieulafoy lesion in the rectum (Figure 1). Four through-the-scope clips were applied to the lesion and injected with a 6 mL 1:1000 solution of epinephrine (Figure 2). No other lesions suspicious for recent bleeding were identified on sigmoidoscopy.

The patient stayed in the medical intensive care unit for one more day and was downgraded to the general medical floor as he was hemodynamically stable; his subsequent two hemoglobin readings after the flexible sigmoidoscopy six hours apart were above 10 mg/dL. While on the general medical floor, his hypernatremia was treated with free water flushes, and he became alert and oriented to person, place, and time after his sodium was within normal limits. His acute kidney injury resolved with the fluids he received, and his hemoglobin remained stable above 10 g/dL with no signs of lower gastrointestinal bleeding reported. He was discharged on his 15th hospital day to his new nursing facility. When following up on the patient one week after discharge, the nursing home facility staff denied any signs of a rebleed.

## Discussion

Dieulafoy lesions are dilated submucosal arteries that occasionally are the source of gastrointestinal bleeding. The majority of these lesions are in the proximal stomach along the lesser curvature, commonly near the esophagogastric junction [1]. Endoscopic intervention with esophagogastroduodenoscopy is usually the first line for diagnosis, which reveals a pigmented protuberance with minimal surrounding erosion without ulceration (visible vessel sans ulcer) [2]. Our patient had significant bright red blood per rectum resulting in a substantial decrease in hemoglobin and hemodynamic instability. This encounter is a rare finding as these lesions are most commonly associated with upper gastrointestinal bleeding. A few published case reports demonstrate this finding in the colon, and 37 case reports demonstrate this lesion in the duodenum [3-5].

There are also additional case reports that also present with patients having these lesions in their rectum. One report by Then et al. [6] describes a patient with a similar presentation to the one in this case who had a sharp drop in hemoglobin that led to a colonoscopy. Hemostasis was achieved similarly with three hemostatic clips and an epinephrine injection to the lesion site. In another report by Paz et al. [7], their patient also had a sharp drop in the red blood cell count, was hypotensive, and required to be upgraded to the medical intensive care unit for closer monitoring of his vitals. The patient had a bleeding rectal Dieulafoy lesion, and the area received hemostatic clips and epinephrine injection. In both cases, adrenaline injection and through-the-scope clips helped achieve hemostasis.

A significant issue in treating gastrointestinal bleeds is the risk of rebleeding. One study by Massinha et al. [8] found that 73 hospitalized patients with a Dieulafoy lesion were actively bleeding and treated with a combination of adrenaline injection, hemoclips, or argon plasma to achieve hemostasis; 12 patients had an episode of rebleeding during the initial hospitalization. This study also showed that patients under antiplatelet therapy showed signs of early relapse [8]. Standard endoscopic treatment methods for treating an actively bleeding Dieulafoy lesion include endoclips, epinephrine injection, and thermal coagulation. One study found similar initial hemostasis rates between the endoclips to epinephrine injections. However, there were significantly lower rebleeding rates in endoclips, which was 0%, to epinephrine injection, 35% [9]. Another study found similar findings, where the rate of rebleeding was 8.3% with endoscopic clips versus 33% with injection therapy alone [10]. Based on these studies, it appears that hemostatic clips are more superior to injection therapy alone in preventing rebleeding. Through-the-scope clips and over-the-scope clips are two different types of endoclips, and there are no published randomized comparisons of the two in terms of superiority for treating bleeding Dieulafoy’s lesions yet.

Based on these case reports, although relatively rare and, when present, usually located in the stomach, it appears that these lesions are becoming more prevalent in other regions of the gastrointestinal tract. Although there is still no clear etiology toward the cause of these lesions, studies have shown that they are found primarily in men with cardiovascular disease, chronic kidney disease, chronic non-steroidal anti-inflammatory drug (NSAID) use, and alcohol abuse [11].

Healthcare professionals should be keen on preventing gastrointestinal bleeding in the elderly population, especially Dieulafoy lesions, given how acutely these lesions can lower a patient’s hemoglobin, such as in this case. One solution would be to limit the prescription of NSAIDs to patients, especially the elderly population. Most patients are on daily aspirin for coronary artery disease to prevent cardiovascular complications such as acute coronary syndrome. However, the aspirin in reducing events in the elderly (ASPREE) primary prevention of cardiovascular disease trial showed that patients aged above 70 years who take low-dose aspirin therapy have increased upper gastrointestinal bleeding without the significant cardiovascular benefit [12]. COX-2 inhibitors have been the alternative to NSAIDs as this class of medications does not interfere with COX-1, which protects the gastric mucosa [13]. One possible concern with this alternative is its potential to delay the healing of gastric erosions or ulcers, although these clinical trials did not show a significant delay in ulcers for patients taking celecoxib [14].

Patients' history should be reviewed before prescribing NSAIDs or COX-2 inhibitors chronically, including a history of adverse gastrointestinal events, concurrent use of low-dose aspirin, and age greater than 64. One prospective study shows that these factors are significant predictors of gastrointestinal toxicity, including bleeding, perforation, obstruction, or uncomplicated ulcer [15]. Given the arterial nature of the bleed, Dieulafoy lesions can result in profuse bleeding and associated hemodynamic instability, which can result in severe morbidity and mortality if left untreated.

## Conclusions

This case aims to highlight an uncommon finding as a potential source of acute lower gastrointestinal bleeding. Although unusual outside the stomach, it can be a source of acute blood loss from a lower gastrointestinal bleed. The onset that patients can become hemodynamically unstable with an active bleed from a Dieulafoy lesion, as seen in this patient, is rapid and needs immediate endoscopic intervention with hemostatic clipping as it is more superior to epinephrine injections alone in preventing rebleeding. Patients with cardiovascular disease, chronic kidney disease, chronic NSAID use, and alcohol abuse are more likely to develop these lesions. Primary care physicians should make goals to help mitigate the progression of their chronic conditions, limit NSAID use, and reduce alcohol consumption to prevent these patients from developing Dieulafoy lesions.
